# An Integrated Framework for Automated Image Segmentation and Personalized Wall Stress Estimation of Abdominal Aortic Aneurysms

**DOI:** 10.3390/bioengineering13020191

**Published:** 2026-02-07

**Authors:** Merjulah Roby, Juan C. Restrepo, Deepak K. Shan, Satish C. Muluk, Mark K. Eskandari, Vikram S. Kashyap, Ender A. Finol

**Affiliations:** 1 Department of Mechanical, Aerospace, and Industrial Engineering, The University of Texas at San Antonio, San Antonio, TX 78249, USAender.finol@utsa.edu (E.A.F.); 2 Department of Thoracic and Cardiovascular Surgery, Allegheny Health Network, Allegheny General Hospital, Pittsburgh, PA 15212, USA; 3 Feinberg School of Medicine, Northwestern University, Chicago, IL 60611, USA; 4 Cardiovascular Health, Corewell Health, Grand Rapids, MI 49503, USA; vikram.kashyap@corewellhealth.org

**Keywords:** abdominal aortic aneurysm, deep learning, image segmentation, computed tomography imaging, Non-Uniform Rational B-Splines, wall stress, nonlinear elastic membrane analysis

## Abstract

Abdominal Aortic Aneurysm (AAA) remains a significant public health challenge, with an 82.1% increase in related fatalities from 1990 to 2019. In the United States alone, AAA complications resulted in an estimated 13,640 deaths between 2018 and 2021. In clinical practice, computed tomography angiography (CTA) is the primary imaging modality for monitoring and pre-surgical planning of AAA patients. CTA provides high-resolution vascular imaging, enabling detailed assessments of aneurysm morphology and informing critical clinical decisions. However, manual segmentation of CTA images is labor-intensive and time consuming, underscoring the need for automated segmentation algorithms, particularly when feature extraction from clinical images can inform treatment decisions. We propose a framework to automatically segment the outer wall of the abdominal aorta from CTA images and estimate AAA wall stress. Our approach employs a patch-based dilated modified U-Net model to accurately delineate the outer wall boundary of AAAs and Nonlinear Elastic Membrane Analysis (NEMA) to estimate their wall stress. We further integrate Non-Uniform Rational B-Splines (NURBS) to refine the segmentation. During prediction, our deep learning architecture requires 17±0.02 milliseconds per frame to generate the final segmented output. The latter is used to provide critical insight into the biomechanical state of stress of an AAA. This modeling strategy merges advanced deep learning architecture, the precision of NURBS, and the advantages of NEMA to deliver a robust and efficient method for computational analysis of AAAs.

## 1. Introduction

Abdominal Aortic Aneurysm (AAA) is a serious condition in which the abdominal segment of the aorta dilates abnormally. If it ruptures, the mortality rate can be up to 80%. The probability of developing an AAA increases with age, smoking, high blood pressure, plaque formation (atherosclerosis), and male sex [1,2]. AAAs often do not present symptoms in their early stages, making detection difficult without screening. However, as the aneurysm expands, it can cause severe abdominal or back pain. In clinical practice, vascular surgeons or interventional radiologists use the maximum diameter of the aneurysm to estimate its future risk of rupture [3]. Current guidelines recommend surgery for men if the aneurysm is at least 5.5 cm in diameter and for women if it is in the range of 5.0 to 5.4 cm in diameter [2].

Several advances have been made in the development of clinical image segmentation methods to identify aortic aneurysms. Kurugol et al. [4] used unenhanced computed tomography angiography (CTA) images to quantify the shape of an AAA by combining anatomical localization, circular Hough transform, and 3D level set segmentation. Shang et al. [5] utilized a semi-automated method to monitor the AAA wall by recognizing isointensity contours in CTA images. De Bruijne et al. [6] proposed manual delineation followed by automatic segmentation of CTA images using a statistical shape model. Wang et al. [7] improved the geodesic active contour approach to segment AAAs using MRI data. Herment et al. [8] proposed a 2D deformable surface model to segment the thoracic aorta in MR images. Xie et al. [9] created an algorithm that uses anatomical localization and cylinder tracking to segment the thoracic aorta in unenhanced CTA images.

Before deep learning techniques were developed, semiautomated approaches for image segmentation were implemented. Although these methods yielded reasonable results, they often required extensive fine-tuning to achieve optimal precision. Given the limitations of the aforementioned approaches, the need for automated segmentation tools for medical image analysis is warranted. Chandrashekar et al. [10] used deep learning to automatically segment aortic aneurysms in CTA images, achieving DSC (Dice Similarity Coefficient) scores of 0.887 ± 0.005 at low resolution and 0.932 ± 0.007 at high resolution. Lyu et al. [11] compared the performance of two advanced models, ARU-Net and CACU-Net, and found that they achieved DSC scores of 0.916 ± 0.028 and 0.916 ± 0.029, respectively. Kim et al. [12] used a convolutional neural network (CNN)-based segmentation technique (developed by Zhang et al. [13]) to identify the lumen and outer wall from 3D CTA scans, resulting in a DSC score of 0.866 ± 0.044. These techniques have significantly improved AAA segmentation and although future tests on larger and more complex cases will help demonstrate their effectiveness in clinical practice, models must be fine-tuned to improve their accuracy and reliability.

As an alternative risk assessment surrogate beyond the maximum diameter, peak wall stress can be estimated using 3D geometries generated after image segmentation using Finite Element Analysis (FEA) [14]. However, predefined constitutive material properties are required as input to traditional forward FEA simulations. These material properties are typically derived from uniaxial and biaxial tensile tests of ex vivo tissue samples, with material models based on assumptions about different biomechanical behaviors, such as isotropic or anisotropic characteristics in elastic or hyperelastic material frameworks [15]. Despite a large sample base, material behavior can vary in vivo and regionally on a subject-specific basis, potentially leading to inaccurate wall stress estimates. For example, the tensile strength of ruptured AAA tissue was found to be lower than that of the tissue obtained from electively repaired AAAs (54 ± 6 N/cm^2^ vs. 82 ± 9.0 N/cm^2^) [16]. For patients in surveillance programs, it is not yet possible to characterize AAA tissue non-invasively. In contrast, alternate solutions have been proposed to overcome the limitation of the predefined material properties required for FEA. Abdominal/pelvic CTA images represent a deformed configuration of the abdominal aorta, commonly under mean arterial pressure in which static determinancy is maintained. Inverse methods [17,18,19,20,21,22] have been developed to calculate wall stress based on this deformed configuration with known applied loads via membrane equilibrium, which eliminates the need to specify material properties for the AAA wall [23,24].

The primary objective of this work is to develop a framework using a novel image segmentation strategy that meets the demand for automatic segmentation and precision integrated with a wall stress estimation procedure. Combined, these methods enable the creation of AAA models that are used to estimate AAA wall stress using a non-linear elastic membrane model without user intervention, overcoming the need for pre-defined material properties.

## 2. Methods

### 2.1. Study Subjects

We retrospectively collected 147 CTA exams with a slice thickness of 1–3 mm from 76 symptomatic and 71 asymptomatic patients at Allegheny Health Network and Northwestern Memorial Hospital, after approval of Allegheny Health Network’s Institutional Review Board (IRB) at Allegheny General Hospital (Pittsburgh, PA, USA) and Northwestern University’s IRB at the Feinberg School of Medicine (Chicago, IL, USA). All methods were performed in accordance with the relevant guidelines and regulations of the respective IRBs. Consequently, informed consent was waived by both IRBs for this retrospective study of existing de-identified clinical data. The data set includes 9037 images, which were semi-automatically segmented with the in-house tool AAAVasc, validated previously [25]. The data set represents ground-truth data for training and testing the accuracy of the framework’s automated segmentation algorithm. In addition, we used 35 CTA data sets from 12 different AAA participants in a surveillance program to apply the framework for the prediction of wall stress.

### 2.2. Segmentation of the AAA Outer Wall

We based the 2D U-Net architecture on Ronneberger’s et al. [26] original U-Net model, optimizing it specifically for CTA image segmentation. Its encoder-decoder construction resembles a “U,” which helps in the extraction of detailed features and the generation of high-quality output. In this architecture, dilation rates are critical to how the network interprets images in its convolutional layers. These rates describe the spacing between pixels in convolutional filters. A higher dilation rate indicates the filters are farther apart, allowing them to gather information from a larger area of the image. The effective receptive field of a dilated convolution is defined by Equation (1),

(1) RF=(k−1)·d+1

where *d* represents the dilation rate and *k* the kernel size. In a conventional CNN layer, convolutions use a fixed d=1, which means that each kernel works on adjacent pixels. This limits the receptive field; to capture a larger context, deeper networks or pooling mechanisms are required. In contrast, dilation enables the network to detect long-range correlations while maintaining spatial resolution by increasing the receptive area without introducing new parameters. This is particularly useful in medical image segmentation, where exact identification of minute anatomical details is critical.

**Figure 1:**
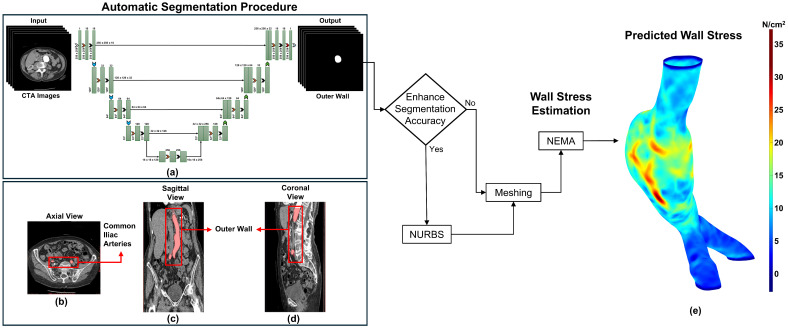
Framework for automated segmentation and personalized wall stress in abdominal aortic aneurysms (AAAs): (**a**) Architecture of the patch-based dilated U-Net model; (**b**) Exemplary binary masks overlaid on an AAA image; (**c**,**d**) Reconstruction and visualization of the AAA in the coronal and sagittal planes; (**e**) First principal wall stress distribution in a mesh generated using the automated segmentation; stress values are in N/cm^2^.

In our U-Net design, we intentionally alter the dilation rates between layers. We begin with a dilation rate of one, meaning that the filters scrutinize each pixel closely. Subsequently, we increase the rates to 2 and 3, before returning to 1. By gradually changing the dilation rate, our model identifies both local and global data more effectively than typical CNN designs, resulting in higher segmentation accuracy. This method is beneficial for feature extraction, since it enables the network to detect and analyze complicated patterns and structures in the image. Figure 1a illustrates the overall study design of the U-Net architecture for clinical image segmentation. In addition to architectural components, our methodology includes patch-based processing. This technique breaks large image stacks into smaller, more manageable patches that are processed individually. It offers quick analysis and segmentation of large data sets, allowing complete analysis while managing computing complexity. For training, the model was optimized using the Adam optimizer with an initial learning rate of $1\times 10^{-4}$. We coupled it with a ReduceLROnPlateau scheduler that decreased the learning rate by a factor of 0.5 after five consecutive epochs without improvement in the validation DSC (minimum learning rate $1\times 10^{-6}$). A batch size of 16 was used and early stopping with a patience of 5 epochs was applied to prevent overfitting. Training typically converged at approximately 219 epochs. To obtain the best border delineation, the segmentation model is trained using a combination of the Dice loss function and binary cross-entropy (BCE) loss, which balances pixel-wise classification confidence (BCE loss) with area overlap accuracy (Dice loss). Furthermore, data augmentation procedures are used to expand the training data set and improve the generalization of the model. Augmentation techniques such as horizontal and vertical flipping, rotation, intensity scaling, and zoom transformations were applied to make the model more adaptable and accurate when analyzing new unseen images. It also reduces overfitting by using several representations of the training data, improving the ability to handle additional unknown data during inference. Additional methodological details are provided elsewhere [27,28]. The inference time was measured using PyTorch 2.5.1 (CUDA 12.1) with the torch.cuda.Event mechanism on an NVIDIA RTX A6000 GPU (47.5 GB of dedicated memory and 111 GB of total memory).

### 2.3. User-Interactive NURBS-Based Tool

We created a user-friendly tool using non-uniform rational basis splines (NURBS) [29], which was developed as an interactive Python 3.9 application with a graphical user interface created using Tkinter. It enables users to input CTA images and their related segmentation masks, and manually fine-tune segmentation boundaries by modifying interactive points on the contours. This real-time user intervention can be critical for medical experts who need to make rapid changes so that the contours accurately represent their anatomical features of choice. Creating a NURBS curve using control points requires calculating a knot vector, which affects the manner the curve transitions between points. The curve is then evaluated at particular locations along its length using Equation (2),

(2) $$c\left(t\right)=\frac{\sum_{i=0}^{n}N_{i,p}\left(t\right)w_{i}P_{i}}{\sum_{i=0}^{n}N_{i,p}\left(t\right)w_{i}}$$

where c(t) is the point on the NURBS curve at parameter *u*; $N_{i,p}\left(t\right)$ is the ith B-spline basis function of degree *p*; $w_{i}$ is the weight associated with the ith control point; $P_{i}$ is the ith control point; and *t* is the parameter, which varies from 0 to 1 along the length of the curve. In this approach, we replace the original boundary with a smooth curve drawn through evenly spaced points using a fixed number of points and curve order. The result is then converted back into a binary mask for comparison with the original segmentation.

The NURBS tool allows users to manually fine-tune image segmentation by modifying contour points and adding new ones as necessary. It also offers a technique for improving segmentation masks using smooth curves, which yield more precise shapes. Users can proceed with NURBS-based changes or revert to automatic segmentation results. The technique enables users to make informed decisions about how automated forecasts or NURBS modifications may affect future treatment suggestions.

### 2.4. Nonlinear Elastic Membrane Analysis (NEMA) for Wall Stress Estimation

Pressure vessels are nearly statically determined even when they have thick walls [30]. Therefore, intramural stress depends only on the geometry and internal load, allowing AAA wall stress to be determined by solving for elastic determinancy [17]. This approach enables the estimation of wall stress without relying on predefined material properties. In this analysis, the equilibrium equations were solved for a deformed configuration of the AAA. Using linear shape functions to represent the spatial variation of the components of the equilibrium equation, i.e., Equation (3), effectively yields a set of non-linear algebraic equations [21] in $\sigma^{\alpha \beta }$. Consequently, the components of the Cauchy stress tensor can be evaluated at the centroid of each element [17,18,24] and the aforementioned algebraic equations are solved using the inverse method:

(3) $$\frac{1}{\sqrt{g}}{\left(\sqrt{g}\;h\;\sigma^{\alpha \beta }\;g_{\alpha }\right)}_{,\beta }+p\;n=0$$

where *g*: determinant of the metric tensor; *h*: element thickness; $\sigma^{\alpha \beta }$: Cauchy stress components to be determined in the local contravariant basis (α,β=1,2); and *p*: intraluminal pressure.

NEMA takes as input a 3D triangular surface mesh representing the outermost layer of the wall in its deformed configuration—under mean arterial pressure—with a specified wall thickness. Intraluminal pressure was applied outwards to the internal faces of the mesh elements, while the distal and proximal ends were fixed. These 3D meshes were constructed using the AAAMesh custom script [31] using CTA-specific slice spacing and pixel size. NEMA was previously validated by Thirugnanasambandam et al. [32] using a benchtop flow loop with a deformable AAA silicone phantom of a patient-specific geometry. The phantom was imaged using MRI to acquire time-resolved images of the wall. From these images, a 3D geometry was generated at a peak systolic pressure of 140 mmHg, which was the internal load for NEMA. The results were compared to those of an FEA simulation using an isotropic linear elastic material on the zero-pressure geometry derived from the MRI data [24].

In this work, we applied NEMA to predict the first principal wall stress for 105 surface meshes from 12 AAA patients under surveillance (derived from 35 CTA exams) in three groups: Ground Truth, Predicted, and Predicted + NURBS. Each mesh had a uniform wall thickness of 1.5 mm and was subjected to a mean arterial pressure of 93.3 mmHg with fixed proximal and distal ends. This pressure was estimated from systolic and diastolic pressures measured in a healthy individual (120/80 mmHg). However, this assumption may not always be reliable for AAA patients, who frequently have hypertension [33]. Figure 1 shows the integrated framework for automated segmentation and personalized wall stress estimation in AAAs. Figure 1b–d shows an exemplary result of the outer wall boundaries of an AAA. Figure 1b displays the axial plane of an initial CTA image overlayed with the binary mask, while Figure 1c,d depict the coronal and sagittal planes, respectively. Figure 1e shows the first principal wall stress distribution predicted using NEMA.

## 3. Results

### 3.1. Identification of the Outer Wall Boundary

**Figure 2:**
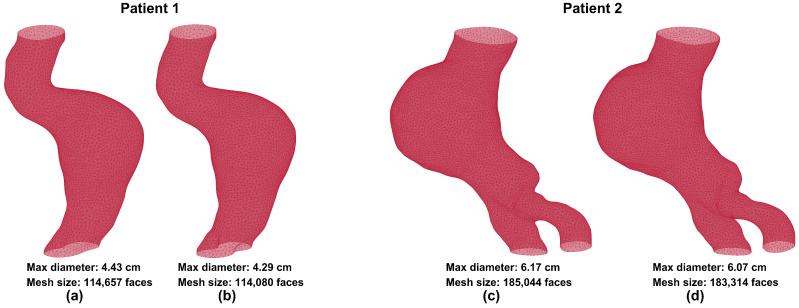
Volume meshes generated from the ground truth (**a**,**c**) and the predicted (**b**,**d**) image segmentations.

The outer wall boundary was detected accurately using the patch-based dilated U-Net model, which was trained on 9037 contrast-enhanced CT images and tested on an additional 1063 images. To assess segmentation accuracy, the output of the model, i.e., the predicted outer wall boundaries, was compared to the ground truth obtained from previously segmented images using AAAVasc. Figure 2 shows visual comparisons of volume meshes generated from ground truth and predicted segmentations for two male patients. Patient 1 was 71 years old and patient 2 was 76 years old at the time of imaging. Figure 2a,c show the ground truth volume meshes, while Figure 2b,d show the predicted volume meshes, respectively. The maximum diameters and mesh sizes are reported for each mesh in the figures. This comparison illustrates the qualitative differences and similarities that are evident by visual inspection of the 3D geometries resulting from the ground truth and predicted segmentations.

### 3.2. Performance Metrics for Segmentation Assessment

We used eight metrics to evaluate the performance of the U-Net segmentation model computed using the test dataset of 1063 images to assess the efficiency of the model on unseen data. The model identified and delineated the AAA boundaries of interest for the outer wall segmentation with high accuracy, sensitivity, and precision, as shown in Table 1.

The MCC and DSC indicate strong agreement between the predicted and ground-truth segmentations, while the specificity shows effective exclusion of non-relevant regions in the segmentation ROIs. The IoU further reflects accurate outer wall segmentation and robust handling of the anatomical components of the abdominal aorta. The mean 95% HD quantifies the maximum boundary deviation between the predicted segmentation and the ground truth, with the 95% HD commonly used in medical imaging to reduce the influence of outliers by reporting the 95th percentile of distances. Additional metrics and a comparison with other segmentation methods were reported in [28].

### 3.3. Maximum AAA Diameter

**Figure 3:**
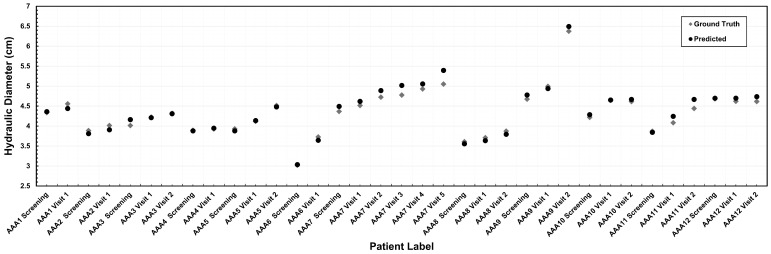
Comparative analysis of the maximum hydraulic diameter (in cm) measured using the outer wall boundary.

We evaluated the segmentation model’s accuracy using the maximum diameter to assess suitability for a clinical application. This was performed by comparing the maximum hydraulic diameter obtained using two different segmentation methods: expert medical segmentation (ground truth) and predictive algorithmic segmentation. Figure 3 illustrates this comparison for the 12 surveillance patients at each of the CTA imaging follow-ups (initial screening and one or more subsequent visits), which produced 35 measurements of maximum diameter. The absolute difference in maximum diameter between predicted and ground truth measurements was 0.09 ± 0.07 cm, with values ranging from 0.008 cm to 0.34 cm.

### 3.4. Wall Stress Estimation

**Figure 4:**
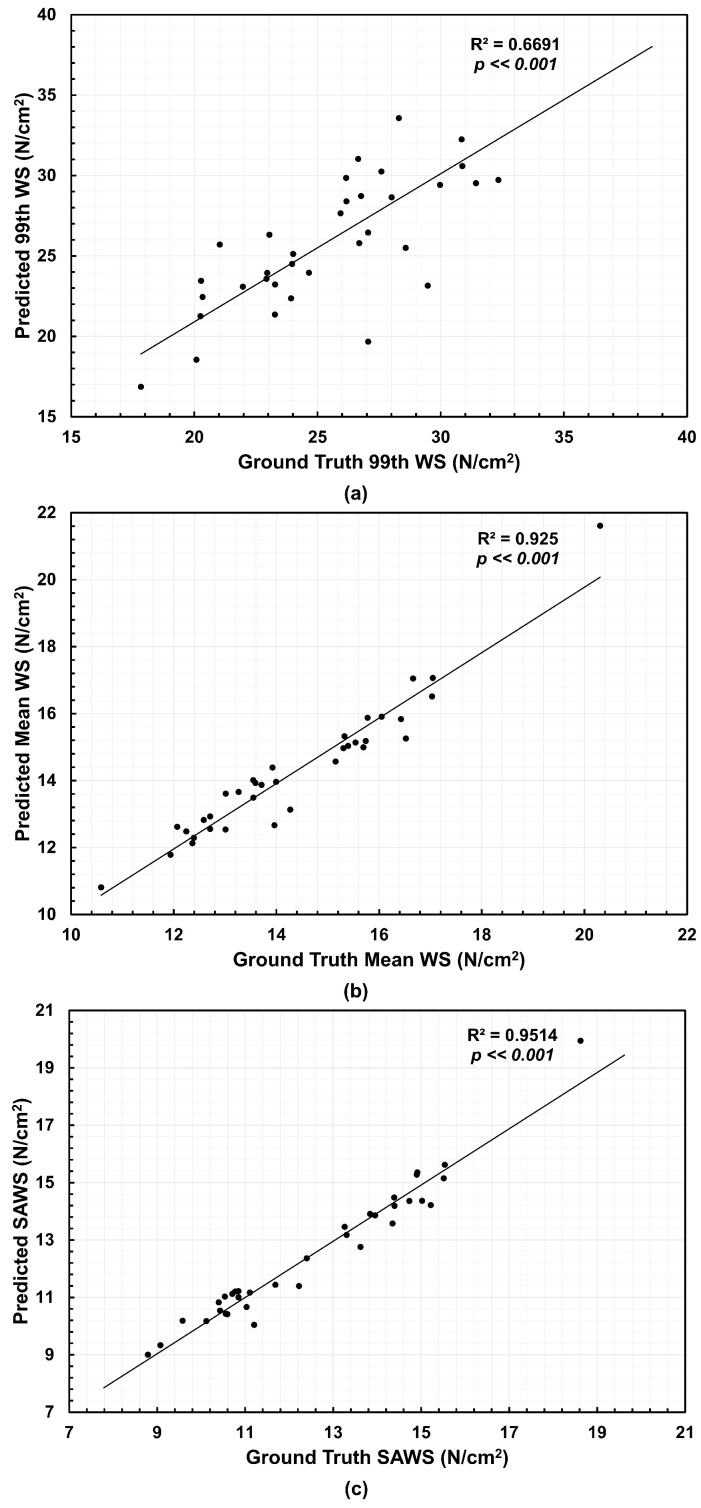
Correlations relative to the Ground Truth and their coefficients of determination ($R^{2}$) for the (**a**) 99th percentile wall stress (99th WS), (**b**) mean wall stress (Mean WS), and (**c**) spatially averaged wall stress (SAWS), using the predicted meshes (*n* = 35 data points, which corresponds to 12 AAA patients under surveillance).

From the NEMA stress distributions, three biomechanical parameters were calculated, namely, 99th percentile wall stress (99th WS), mean wall stress (Mean WS) and spatially averaged wall stress (SAWS). The latter was calculated as the area-weighted average of wall stress using the surface areas of the mesh elements. We compared the predicted wall stress relative to the ground truth wall stress using the coefficient of determination $R^{2}$. Figure 4 shows the correlations for the meshes generated directly from the U-Net segmentations and Ground Truth. The 99th WS achieved an $R^{2}$ of 0.6691, the Mean WS reached an $R^{2}$ of 0.925, and SAWS resulted in an $R^{2}$ of 0.9514 when compared to their respective ground truth counterparts. We observed a higher dispersion for the 99th WS with over- and under-estimations. For Mean WS and SAWS, nearly half of the 35 AAA geometries underestimated these biomechanical parameters.

**Figure 5:**
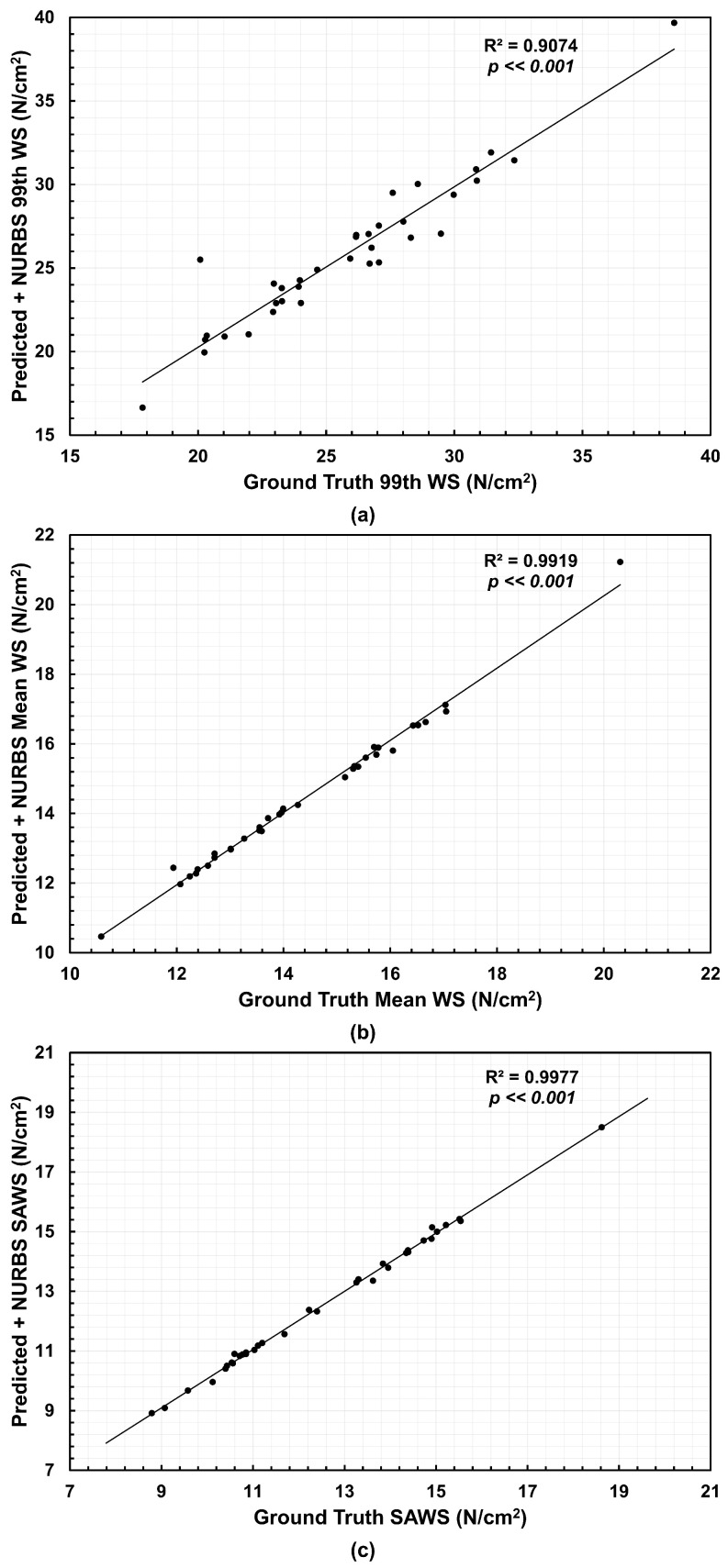
Correlations relative to the Ground Truth and their coefficients of determination ($R^{2}$) for the (**a**) 99th percentile wall stress (99th WS), (**b**) mean wall stress (Mean WS), and (**c**) spatially averaged wall stress (SAWS) using the NURBS-corrected predicted meshes.

The effect of applying the NURBS-based correction is shown in Figure 5. This adjustment improved the agreement for all three parameters. The 99th WS yielded an $R^{2}$ of 0.9074, the Mean WS an $R^{2}$ of 0.9919, and SAWS an $R^{2}$ of 0.9977. These results indicate that the corrected meshes reproduce the ground truth stress distributions with high fidelity, improving the accuracy compared to the predicted meshes (without NURBS-based correction) alone.

**Figure 6:**
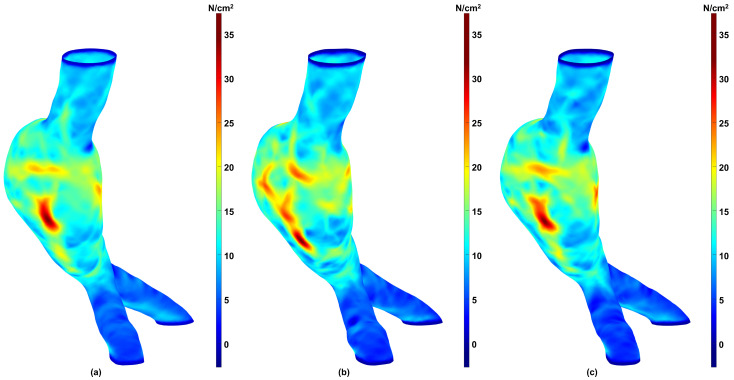
First (maximum) principal wall stress distribution for an exemplary AAA representative of the Ground Truth (**a**), Predicted (**b**), and Predicted + NURBS (**c**) groups. Stress values are in N/cm^2^.

To evaluate whether the differences in biomechanical parameters obtained for the three aforementioned groups were statistically significant, we performed a Friedman test (Friedman Statistic, FS) with a significance level of α=0.05 using GraphPad Prism version 10.6.1 for Windows (GraphPad Software, Boston, MA, USA, www.graphpad.com). This approach enabled pairwise comparisons between the Predicted and Predicted + NURBS groups against the Ground Truth group. The Friedman test revealed that there are no statistically significant differences between the groups for any parameter: 99th WS (FS =4.171, p=0.1242), Mean WS (FS =1.086, p=0.5811), and SAWS (FS =1.086, p=0.5811). The relative similarity revealed by the Friedman test can be observed using a stress map on the surface meshes. Figure 6 shows color maps of the spatial distribution of the first principal wall stress for an exemplary AAA geometry using (a) the Ground Truth mesh; (b) the mesh generated directly from the predicted segmentation; and (c) the mesh generated after applying NURBS correction to the predicted segmentation.

## 4. Discussion

In this study, we developed a deep learning–based model to automatically segment the outer wall of AAAs for biomechanical analysis. To demonstrate an additional feature of the framework, an interactive post-processing step was used to further refine the automatically generated segmentation results. The interactive NURBS-based tool improves the accuracy of the model, which is especially useful to allow user intervention if desired. Performance metrics reveal that the model is highly accurate, suggesting its potential for use in a clinical setting as part of a risk assessment modeling pipeline. Beyond the maximum diameter, rupture risk assessment is performed with methods such as vascular deformation mapping (VDM) and wall stress estimation. VDM quantifies regional, three-dimensional growth from serial CTAs [34], whereas stress estimators (e.g., NEMA or FEA) provide single time-point biomechanical loading. Our study focuses on wall stress estimation using NEMA, which requires only the outer wall geometry. Therefore, we limited the use of the segmentation module to the outer wall boundary, although it is capable of segmenting the lumen and intraluminal thrombus, which are not needed for the subsequent biomechanical analysis with NEMA. The output of the segmentation model was subsequently used to create in-silico models of 12 AAA patients under surveillance with multiple imaging follow-ups. These patient-specific models provide an evaluation of the biomechanical state of each AAA over time, which in turn can be used by the treating physician to make an informed decision about recommending AAA repair.

**Figure 7:**
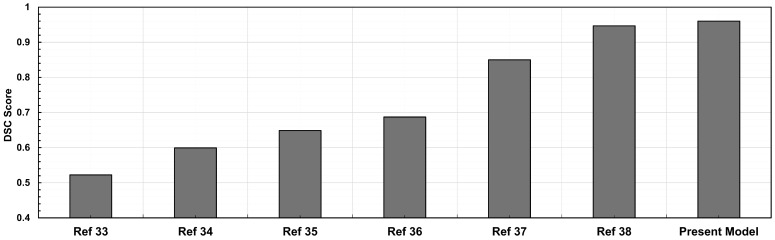
Comparative analysis of CTA image segmentation models based on DSC scores: existing models vs. present model.

The proposed patch segmentation method has several advantages compared to existing automatic segmentation approaches. The traditional deep learning models use standard convolutions with a confined receptive field; our model’s dilated convolutions efficiently enhance the receptive field without increasing processing costs. This enables more precise segmentation of the anatomical structures for improved feature extraction. Furthermore, unlike existing segmentation algorithms, which often process complete images at once, our patch-based methodology provides greater resolution in feature extraction by breaking down images into smaller ones. The accuracy of the model, with a DSC of 96.95%, outperforms existing CNN-based techniques [27]. Although Roby and colleagues used a different data set, our model provides greater segmentation reliability, reducing false positives and false negatives, which we infer would have translational relevance. The fully automated segmentation pipeline predicts an image in 17 ± 0.02 milliseconds, eliminating the need for manual correction. This greatly reduces processing time compared to existing approaches that require manual adjustment or additional fine-tuning steps. In cases with highly calcified aneurysm walls, calcium deposits increase pixel intensity differentiation with surrounding tissues, which helps the model detect the outer wall boundary more clearly. We recognize partial volume effects as a limitation of CT imaging. While the model does not directly correct them, their impact is minimized through patch-based training, intensity and scale augmentations, and, when necessary, manual refinement with the NURBS tool. Figure 7 compares DSC values from our proposed method with those reported for existing segmentation approaches. The methods differ in model design, training setup, preprocessing steps, and datasets, as reported in the existing study [35,36,37,38,39,40].

Lareyre et al. [41] developed an automated pipeline for AAA lumen segmentation employing feature-based techniques, including boundary propagation and active contour methods. Although their method performed well on a data set of 40 CTAs, it produced false positives and negatives, especially when identifying the aorta and renal arteries. Their pipeline is time efficient, while segmenting the lumen in less than 1 min per patient. In contrast, the implementation of our NURBS-based tool for users to interactively modify segmentation boundaries results in anatomically correct representations of the aortic wall based on the user’s choice. The NURBS-enhanced model reduced image processing time by 90% compared to the ground-truth method, namely the segmentation provided by AAAVasc, making it more appealing for real-time clinical applications where speed and accuracy are important.

The comparative analysis of three biomechanical parameters for the three groups revealed quantifiable differences in wall stress estimates. For example, we observed a higher dispersion for the 99th WS in contrast to the Mean WS and SAWS, as shown in Figure 4 for the predicted meshes and Figure 5 when NURBS correction is used. However, these differences were not found to be statistically significant. We infer from this result that the automated segmentation model with or without NURBS corrections yielded in-silico AAA models that were statistically similar to the Ground Truth for wall stress estimation (for the surveillance AAA population used in this work). Nevertheless, while the differences in the global biomechanical parameters may be statistically insignificant between the three groups, the spatial distributions of wall stress are not identical. As shown in Figure 6, the wall stress distribution of the Predicted + NURBS model closely matches that of the Ground Truth model. This outcome appears to indicate that NURBS refinement of the outer wall segmentation effectively enhances the accuracy of the predictive model compared to an established ground truth observation, albeit at the cost of additional time devoted to NURBS refinement. It should be noted that our geometry-driven NEMA stress estimator is discretization dependent. Even if Ground Truth, Predicted, and Predicted+NURBS segmentations may appear identical, the point-cloud → meshing/smoothing → NEMA pipeline can produce subtly different meshes (leading to different number of nodes, face connectivity and nodal coordinates), which in turn lead to small but measurable differences in global biomechanical parameters, as observed in Figure 4. Therefore, such differences may be confounded by distinct spatial discretizations and not entirely due to differences in outer wall boundary segmentations.

Recently, Chung et al. [42] developed a framework to segment CTA images and estimate wall stress in AAAs. Their model was trained on 400 images from 10 AAAs to identify the lumen and outer wall boundaries, achieving a segmentation accuracy of 0.9980. In contrast, we used 147 AAAs from two different groups (symptomatic and asymptomatic), resulting in a segmentation accuracy of 0.9996. The output from Chung and colleagues’ model was used to generate a 3D surface mesh and extract morphological features for wall stress prediction using an Extra Trees regression model, with ground-truth wall stresses obtained from FEA. Their method achieved a coefficient of determination of 0.98 resulting from the comparison of the predicted wall stress with the ground truth. A comparison of our Predicted + NURBS SAWS with the Ground Truth SAWS resulted in a coefficient of determination of 0.9977. In contrast to the approach used by Chung and co-workers, we did not use a machine learning algorithm to estimate the wall stress. Instead, we directly solved the force balance equations for the predicted geometry (using NEMA), introducing less uncertainty in the final predictions and overcoming the FEA limitation of requiring patient-specific material properties. The automatic segmentation method described in [42] requires 15 s per AAA, compared to our model’s approximately 1.19 s per AAA (17 milliseconds per image) while using GPUs of similar performance (RTX 2080Ti vs. RTX A4500).

BioPARR is an open source software for the estimation of AAA wall stress and rupture potential index [43]. It uses a procedure similar to that of the present work for the estimation of wall stress, in that it overcomes the need for predefined material properties by applying a very stiff material for the AAA wall and ILT [30]. However, it relies on multiple tools to build a complete pipeline. For example, segmentation is performed semi-automatically and takes approximately 45 min per case, which is substantially longer than our proposed segmentation model. Furthermore, an Abaqus license [44] is required to compute the wall stress. In contrast, NEMA is executed with a MATLAB R2024a (The MathWorks, Natick, MA, USA) script in the final step of our modeling pipeline. The script can be compiled into a standalone executable (e.g., via MATLAB Compiler) that runs with the free MATLAB Runtime, so end users do not require a MATLAB license [45]. Alternative wall stress calculations can be performed with free/open-source FEA solvers (e.g., FEBio) under the same modeling assumptions [46]. Note that when stresses are evaluated on the imaged, pressurized AAA geometry with negligible additional deformation, they are effectively independent of the constitutive material parameters. Thus, either an FEA solver (configured accordingly) or an alternative method similar to NEMA will yield equivalent stresses—the “steel aneurysm” argument [30]. Our work is based on a completely automatic pipeline that can estimate wall stress without statistically significant differences relative to ground truth CTA image segmentation. Furthermore, wall stress is estimated using a NEMA-based approach, which is devoid of predefined material properties (as expected in traditional FEA-based methods) and does not depend on access to proprietary commercial software.

The present work is subject to several important limitations that can limit its translational potential for the assessment of AAA rupture risk. One such limitation is the absence of an estimate of segmentation accuracy based on CTA resolution (pixel size and slice thickness). Future work will aim to address the variability of image resolution on the segmentation performance assessment metrics and incorporate unenhanced images into the training data set as they become available. This is especially critical for AAA patients who may experience mild to severe adverse reactions to contrast media [47] or exhibit renal insufficiency. Furthermore, increasing the training data set to incorporate a more extensive range of clinical presentations in CT exams would help the model perform better in more complex AAA cases. Future efforts will also focus on recognizing the inner wall boundary in abdominal CTA images, a problematic task existing models can only handle with user intervention. Another limitation is the use of a NEMA-based approach for biomechanical analysis. Although conventional stress analysis methods are hindered by unknown patient-specific material properties for the wall and ILT, a NEMA-based estimate evaluates stresses using the imaged, pressurized geometry while neglecting bending stiffness and stress gradients across the wall, which forward FEA can resolve [30]. In addition, in vivo wall stresses depend on local wall thickness, which is rarely discernible from CTA images; assuming a uniform wall thickness introduces uncertainty in the magnitude of wall stresses and the location of the peak wall stress [30,48]. Our model currently applies blood pressure directly to the wall and does not explicitly include ILT, despite evidence that ILT modifies load transfer and can reduce the computed wall stresses in thrombus-laden AAAs [49,50,51]. Stresses are computed from a single non ECG-gated CTA configuration that may not correspond to a phase in the cardiac cycle that matches the assumed intraluminal pressure. Thus, the stresses reported may not be representative of those at mean arterial pressure [52,53]. We assumed a standard mean arterial pressure since patient-specific pressures were not available for all study subjects. This assumption affects the absolute stress field, which should not be interpreted as a patient’s “true” wall stress for the purpose of rupture risk assessment. Nevertheless, under the small additional deformation assumption, NEMA stresses scale predictably with applied pressure and are primarily geometry driven, yielding comparable results for similar geometries even if the absolute pressure differs [30]. Consequently, the present comparison of biomechanical parameters is appropriate to demonstrate the effectiveness of the segmentation method, but should not be interpreted as a definitive estimate of patient-specific wall stress.

## 5. Conclusions

This study addresses the need for automatic and reliable AAA segmentation in abdominal CTA images while generating in-silico AAA models for shape modeling and wall stress estimation without user intervention. Using a patch-based dilated U-Net architecture, our methodology successfully overcomes key limitations of earlier CNN designs. The model accurately identifies the boundary of the AAA outer wall (with an IOU score of 0.9411 and a DSC of 0.9695) and achieves precise segmentation in 17 ± 0.02 milliseconds per image. This combination of precision and speed is especially valuable for clinical decision-making in AAA management. To further enhance the model’s effectiveness, we incorporated a NURBS-based tool, which enables user-driven modification of the outer wall boundary if desired. The combination of an advanced deep learning algorithm with NURBS produces a robust solution for the use of in-silico AAA models for wall stress estimation based on NEMA, which could have high translational potential as part of a rupture risk assessment modeling pipeline. The framework can be easily adapted for the segmentation and biomechanical analysis of other vascular systems, extending its utility beyond AAA biomechanical analysis.

## Figures and Tables

**Table 1:** Performance metrics for outer wall segmentation on the test dataset (1063 images).

Metric	Value (Mean ± SD)
Accuracy (%)	99.96 ± 0.01
Sensitivity (%)	97.28 ± 1.90
Precision (%)	96.69 ± 2.00
Specificity (%)	99.98 ± 0.01
Dice Similarity Coefficient (DSC, %)	96.95 ± 1.00
Intersection over Union (IoU, %)	94.11 ± 1.90
Matthews Correlation Coefficient (MCC, %)	96.95 ± 1.00
95% Hausdorff Distance (HD, mm)	1.84 ± 1.69

## Data Availability

The data set used in this study is not publicly accessible due to IRB regulations at the respective clinical centers. However, it can be obtained by making a reasonable request to co-author E.A.F.
